# A Glucagon-Like Peptide-1 Receptor Agonist Lowers Weight by Modulating the Structure of Gut Microbiota

**DOI:** 10.3389/fendo.2018.00233

**Published:** 2018-05-17

**Authors:** Li Zhao, Yi Chen, Fangzhen Xia, Buatikamu Abudukerimu, Wen Zhang, Yuyu Guo, Ningjian Wang, Yingli Lu

**Affiliations:** Institute and Department of Endocrinology and Metabolism, Shanghai Ninth People’s Hospital, Shanghai JiaoTong University School of Medicine, Shanghai, China

**Keywords:** glucagon-like peptide-1, obesity, gut microbiota, metabolism, type 2 diabetes

## Abstract

In addition to improving glucose metabolism, liraglutide, a glucagon-like peptide-1 receptor agonist, has weight-loss effects. The underlying mechanisms are not completely understood. This study was performed to explore whether liraglutide could lower weight by modulating the composition of the gut microbiota in simple obese and diabetic obese rats. In our study, Wistar and Goto-Kakizaki (GK) rats were randomly treated with liraglutide or normal saline for 12 weeks. The biochemical parameters and metabolic hormones were measured. Hepatic glucose production and lipid metabolism were also assessed with isotope tracers. Changes in gut microbiota were analyzed by 16S rRNA gene sequencing. Both glucose and lipid metabolism were significantly improved by liraglutide. Liraglutide lowered body weight independent of glycemia status. The abundance and diversity of gut microbiota were considerably decreased by liraglutide. Liraglutide also decreased obesity-related microbial phenotypes and increased lean-related phenotypes. In conclusion, liraglutide can prevent weight gain by modulating the gut microbiota composition in both simple obese and diabetic obese subjects.

## Introduction

The rising global rates of type 2 diabetes mellitus (T2DM) and obesity present dramatic economic and social burdens, underscoring the importance of effective and safe therapeutic options (1). Recently, gut microbiota has been found to play a critical role in the establishment and maintenance of human health. A wide range of inflammatory and metabolic diseases have been shown to be associated with microbial imbalance (2–4). For example, Vrieze et al. (3) found that obesity was associated with changes in the abundance, diversity, and metabolic function of the gut microbiota, which mainly presented as a higher abundance of *Firmicutes* and a decreased abundance of *Bacteroidetes* in animal studies (5). In human studies, a relation between an aberrant *Firmicutes*/*Bacteroidetes* (F/B) ratio and obesity was observed (6).

The success of glucagon-like peptide-1 (GLP-1) receptor agonists (GLP-1RAs) in the treatment of T2DM highlighted the gastrointestinal tract as a potential target for diabetes treatment. Many studies had shown that the gut microbiota modulated satiety and glucose homoeostasis by inducing the secretion of GLP-1 (7–12). GLP-1 is an incretin hormone secreted by L cells in the intestine in response to food ingestion (13). It can enhance glucose-induced insulin and suppress glucagon secretion. However, natural intact GLP-1 would be degraded rapidly by enzymatic inactivation by dipeptidyl peptidase-IV (DPP-IV) (4, 13). Therefore, various GLP-1RAs and DPP-IV inhibitors were developed to manage clinical hyperglycemia. Most recently, the GLP-1RA liraglutide has been recognized as a promising anti-obesity agent for its “additional effect” on weight loss in obese and/or diabetic individuals (14, 15). The exact mechanism is attributed to reduced food intake, which resulted from the inhibition of appetite and gastric emptying induced by GLP-1 (13, 16). However, some studies demonstrated that GLP-1 could induce more weight loss than could be achieved by restricting the food intake alone (14, 15). This indicates that there may be other mechanisms underlying the weight-losing effect of liraglutide. The literature showed that changes in gut microbiota also had dramatic influences on lipid metabolism, satiety, and ectopic fat deposition (2, 17). Thus, liraglutide, as a GLP-1 analog, might prevent weight gain by modulating the gut microbial composition. Indeed, a previous study found liraglutide could modulate the composition of the gut microbiota by increasing the lean-related profile, consistent with its weight-loss effect in STZ-induced transiently hyperglycemic mice (4). However, in simple obese and spontaneous T2DM obese subjects, the weight-loss effect of liraglutide associated with structural modulation of gut microbiota remains to be elucidated.

In this study, we used Wistar (euglycemic) and Goto-Kakizaki (GK, spontaneous type 2 diabetic) rats fed with a high-fat diet to explore the relationship between the structural modulation of gut microbiota and the weight-control effect of liraglutide.

## Materials and Methods

### Experimental Design

A total of 32 male Wistar and GK rats (3 weeks old) were bred in a pathogen-free environment (22 ± 2°C) with a 12/12-h light/dark cycle and could free access to food and water. After 1 week of acclimation, these animals were fed with a high-fat diet (HFD: 40% carbohydrate, 20% protein, and 40% fat) for 8 weeks. Then, they were randomly divided into four groups and were subcutaneously injected with liraglutide (Victoza, Novo Nordisk, Denmark) or an equal volume of normal saline (NS) for 12 weeks. Wistar rats are non-diabetic, and GK rats are diabetic. Wistar rats are from the same genetic background as GK rats but with normal glucose levels. These two types of rats were all randomly divided into two groups and assigned with one of the treatments: WN (NS, equal volume, *n* = 8), WL (liraglutide 400 μg/kg/day, *n* = 8), GN (NS, equal volume, *n* = 8), and GL (liraglutide 400 μg/kg/day, *n* = 8).

Fasting body weights and blood glucose levels were measured every 2 weeks. Lipid profiles, including total cholesterol (TC), triglyceride (TG), and low-density lipoprotein (LDL) cholesterol, were assayed at the 0, 4th, and 12th weeks using Siemens Dimension MAX (Siemens Healthcare Diagnostics Inc.). Non-esterified fatty acid (NEFA) levels and fasting insulin (FINS) levels were also determined at the 0, 4th, and 12th weeks using the LabAssayTM NEFA kit (Wako, Japan) and ELISA kit (Shibayaji, Japan), respectively. Insulin resistance and insulin sensitivity were evaluated by the homeostatic model assessment-insulin resistance (HOMA-IR) and the insulin sensitivity index (ISI) using the following formulas: HOMA-IR = FBG × FINS/22.5; ISI = ln[1/(FBG × FINS)] (18). After 12 weeks of treatment, the intraperitoneal glucose tolerance test (IPGTT) and intraperitoneal insulin tolerance test (IPITT) were also conducted. This study was carried out in accordance with the recommendations of the ethical principles in animal research adopted by the Department of Laboratory Animal Science, Shanghai JiaoTong University School of Medicine, Shanghai, China and The protocol was approved by the Animal Experimental Ethical Committee of Shanghai JiaoTong University School of Medicine, Shanghai, China.

### Isotope Infusion

All rats were fasted overnight for approximately 12 h before isotope infusion. After local anesthesia with lidocaine, the lateral tail vein and the tail artery were catheterized for the infusion of tracers and blood sampling, respectively. During the experiments, the rats remained conscious and relaxed. [6,6-2D]-glucose (2 µmol/kg/min) and [U-^13^C]-glycerol (0.84 µmol/kg/min) were constantly infused for 90 min through the tail vein using a Harvard mini infusion pump (Harvard Apparatus, Holliston, MA, USA). [9,10-^3^H]-palmitic acid (1 μCi) was injected at 60 min through the tail vein. During the final 10 min, three blood samples (0.5 mL each) were collected from the tail arterial catheter every 5 min for the quantitation of steady-state glucose and glycerol metabolism. Then, the animals were euthanized under anesthesia with pentobarbital (50 mg/kg) to reduce blood elements in tissues. A strip of gastrocnemius muscle (approximately 13 mm × 3 mm × 1 mm) was promptly obtained and cultured *in vitro* to examine β-oxidation of [9,10-^3^H]-palmitic acid (1 μCi). Tissues were quickly obtained, immersed in liquid nitrogen and then stored at −80°C for further analysis.

### Measurement of Isotope Tracers

Plasma samples were processed to obtain the derivatives of [6,6-2D]-glucose and [U-^13^C]-glycerol as described previously (18). Then, gas chromatography/mass spectrometry (Agilent 5975C, Agilent Technologies) was used to measure enrichment of the derivatives. Ions with mass-to-charge ratios (*m*/*z*) of 319 (unlabeled glucose) and 321 (labeled glucose) were selectively monitored. The peak area 321/319 ratio was calculated, and the corresponding enrichment was determined from standard curves. Similarly, the *m*/*z* 221/218 U-^13^C-glycerol and *m*/*z* 215/212 1,2,3-^13^C-glucose ratios were monitored, and their corresponding enrichment was determined.

Hepatic lipids were extracted using the Folch method, and TG concentrations in the liver were assayed with an ELISA kit (Jiancheng, Nanjing, China). Then, pure TG was isolated using thin layer chromatography. In addition, ^3^H_2_O generated from the process of [9,10-^3^H]-palmitic acid β-oxidation was obtained by removing the lipids with chloroform. Next, ^3^H radioactivity in the TG was determined using liquid scintillation counting (LS6500 Multipurpose Scintillation Counter, Beckman, USA), as previously described (18).

### Calculations

The appearance rates of glucose (Ra_glu_) and glycerol (Ra_gly_) were calculated with the steady-state equation from the respective tracer infusion rates (*F*) and mole percent excess (MPE). In the basal state, hepatic glucose production (HGP) is equal to the Ra_glu_ after an overnight fast. The glycerol gluconeogenesis (GNG) rates could be calculated using the MPE of [1,2,3-^13^C]-glucose and [U-^13^C]-glycerol (18). Hepatic lipogenesis was calculated by dividing the total concentrations of TG by the radioactivity of the corresponding labeled TG. All the relative formulas are shown as follows (19):

Raglu(μmol/kg/min)=Fgly(μmol/kg/min)/[6,6-2D]-glucose MPE,

GNG(%)=[1,2,3-13C]-glucose MPE/[U-13C]-glycerol MPE,

Ragly(μmol/kg/min)=Fgly(μmol/kg/min)/[U-13C]-glycerol MPE−Fgly(μmol/kg/min),

$$\begin{array}{l} \text{The synthetic rates of TG}\left(\text{dpm}*\text{Pr}/\text{mmol}\right)\\ ={\;}^{3}\text{H-TG radioactivity}\left(\text{dpm}\right)/\\ \text{total TG concentration}\left(\text{mmol}/\text{g Protein}\right). \end{array}$$

### Adipose Tissue Morphology

Both visceral (mesenteric) and subcutaneous (inguinal) white adipose tissues (WAT) were fixed in 4% paraformaldehyde and were sliced after being paraffin embedded on a microtome (SLEE, Germany). Multiple sections were prepared and stained with hematoxylin and eosin (HE) and were analyzed under an optical microscope (CKX41, Olympus, Japan) to examine the morphological changes.

### Fecal DNA Extraction and 16S rRNA Gene Sequencing

Fresh feces samples were collected at the end of experiment and were immediately stored at −80°C for analysis. Microbial DNA was extracted from feces using the E.Z.N.A. stool DNA Kit (Omega Bio-Tek, Norcross, GA, USA). The extracted DNA was used as the template to amplify the V3 and V4 hypervariable regions of ribosomal 16S rRNA genes by PCR (95°C for 2 min → 25 cycles at 95°C for 30 s, 55°C for 30 s, 72°C for 30 s → a final extension at 72°C for 5 min). The primers were 338F 5′-ACTCCTACGGGAGG-3′ and 806R 5′-GGACTACVGGGTWT-3′: barcode is an eight-base sequence unique to each sample. All PCR reactions were performed in triplicate with 20 µL of final reaction mixture containing 4 µL of 5× Fast Pfu Buffer, 2 µL of 2.5 mM dNTPs, 0.8 µL of each primer (5 μM), 0.4 µL of FastPfu Polymerase, and 10 ng of template DNA. Amplicons were extracted from 2% agarose gels and purified using the AxyPrep DNA Gel Extraction Kit (Axygen Biosciences, Union City, CA, USA) according to the manufacturer’s instructions and quantified using QuantiFluor-ST (Promega, USA). The purified amplicons were pooled in equimolar quantities and paired-end sequenced (2 × 300) on an Illumina MiSeq platform (San Diego, CA, USA) according to standard protocols.

### Statistical Analysis

Data analyses were conducted with IBM SPSS Statistics, Version 22 (IBM Corporation, Armonk, NY, USA). All data were presented as the means ± SDs, and statistical significance was assessed by one-way ANOVA (LSD). Raw “fastq” files were demultiplexed and quality-filtered using QIIME (version 1.17). The reads that could not be assembled were discarded. Sets of sequences with ≥97% identity were defined as an Operational Taxonomic Unit (OTU) using UPARSE (version 7.1).^1^ And chimeric sequences were identified and removed using UCHIME. The phylogenetic affiliation of each 16S rRNA gene sequence was analyzed by Ribosomal Database Project *Classifier*^2^ against the SILVA 16S rRNA database (SSU123, Max Planck Institute, Germany) with 70% confidence threshold. The Rarefaction curve mainly reflecting the microbial diversity of each sample at different sequencing numbers was constructed by using the microbial diversity index of each sample’s sequencing quantity at different sequencing depths. It could be used to compare the richness, homogeneity, or diversity of species in samples with different amounts of sequencing data and also to illustrate whether the amount of sequencing data for a sample was reasonable. The curves tended to be flat, indicating that the amount of sequencing data were large enough to reflect the vast majority of microbial diversity information. In our study, the sobs index and Shannon index of OUT levels were used to conduct the rarefaction curves and the latter was also called Shannon curves. The Shannon index and Chao index were all alpha-diversity indices. The former mainly reflected the community richness and the latter reflected community diversity. The alpha-diversity indices were calculated by Mothur software packages (version V.1.30.1) and the curves were constructed using R packages (version 3.1.0). Principal coordinate analysis (PCoA) was one of the data dimension reduction analysis methods that were used to study the structure of different microbial communities. Unweighted UniFrac distance-metrics analysis was performed using OTUs for each sample. PCoA was then performed based on matrix-of-distance by R programming language. To compare the relative abundance of microbial taxa between different groups, community bar plot analysis was also implemented by R programming language. And the differences of microbial species between the liraglutide-treated group and controls in Wistar or GK rats were assessed by Wilcoxon rank-sum test. Finally, a heatmap based on Spearman’s correlation analysis was constructed to illustrate the correlations between microbial communities and metabolic parameters. *P* < 0.05 was considered significant.

## Results

### Liraglutide Attenuated Blood Glucose Levels, Suppressed Body Weight Gain, and Improved Insulin Resistance

**Figure 1:**
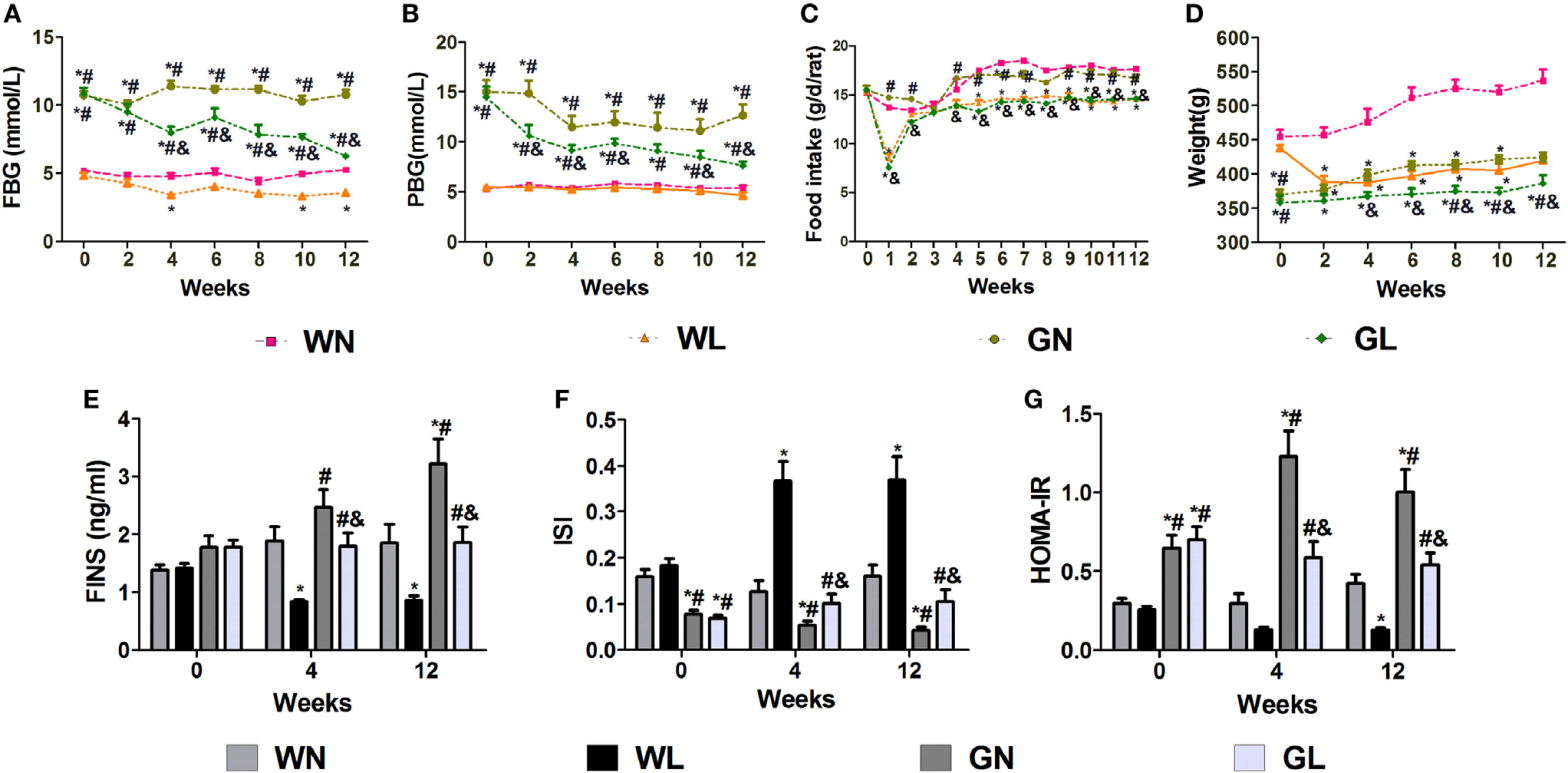
Liraglutide attenuated glucose levels, body weight gain, and insulin resistance. **(A)** Fasting blood glucose (FBG) levels. **(B)** Postprandial blood glucose (PBG) levels. **(C)** Food intake. **(D)** Body weight. **(E)** Fasting insulin (FINS) levels. **(F)** Insulin sensitivity index (ISI). **(G)** The homeostatic model assessment-insulin resistance (HOMA-IR). Data are presented as the mean ± SEM. *P < 0.05 vs WN group; ^#^*P* < 0.05 vs WL group; and ^&^*P* < 0.05 vs GN group.

**Figure 2:**
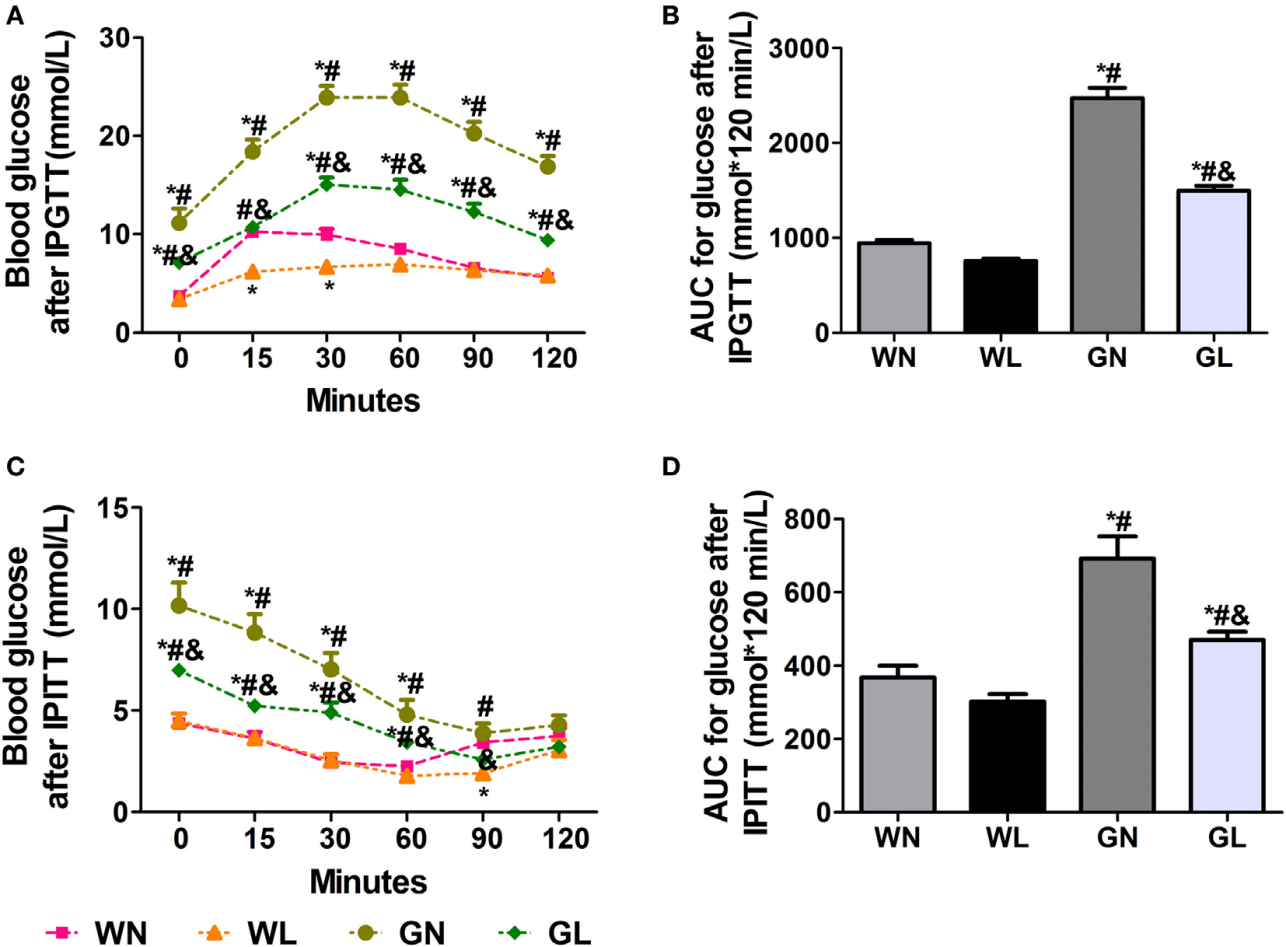
Liraglutide improved glucose tolerance and insulin tolerance. **(A)** Blood glucose levels after intraperitoneal glucose tolerance test (IPGTT). **(B)** The area under the curve (AUC) after IPGTT. **(C)** Blood glucose levels after intraperitoneal insulin tolerance test (IPITT). **(D)** The AUC after IPITT. Data are presented as the mean ± SEM. *P < 0.05 vs WN group; ^#^*P* < 0.05 vs WL group; and ^&^*P* < 0.05 vs GN group.

After the first injection of liraglutide, the anti-hyperglycemic effects were obviously observed in the GK rats within the first 4 weeks, but there was no difference in the two groups of Wistar rats. At the end of experiment, both fasting and postprandial blood glucose levels were significantly decreased in GK rats (Figures 1A,B, *P* < 0.05). The food intake was significantly restricted within the first week after liraglutide treatment in both Wistar and GK rats (*P* < 0.05). From the third week, the food intake of liraglutide-treated groups tended to be stable, but was still significantly lower than the corresponding control groups (Figure 1C, *P* < 0.05). The weight-sparing effects were observed within the first 2 weeks in Wistar rats but within the first 4 weeks in GK rats (Figure 1D). These results manifested that liraglutide was able to attenuate body weight gain without leading to the risk of hypoglycemia. After liraglutide intervention, the improvement of FINS concentrations (Figure 1E) and IR (both ISI and HOMA-IR) (Figures 1F,G) were all observed from the fourth week. After 12 weeks of intervention, the FINS concentrations, the ISI and the HOMA-IR were all greatly improved in the GL and WL groups (*P* < 0.05). Interestingly, these indices had no difference between GL and WN groups, which means the IR was much more serious in GK rats and that a lager dose of liraglutide was needed to improve IR in diabetes patients with obesity. The results of IPGTT (Figures 2A,B) and IPITT (Figures 2C,D) showed that glucose tolerance and insulin tolerance were improved greatly by liraglutide in GK rats rather than in Wistar rats.

### Liraglutide Decreased Lipid Profile and Improved Leptin and Adiponectin Levels

**Figure 3:**
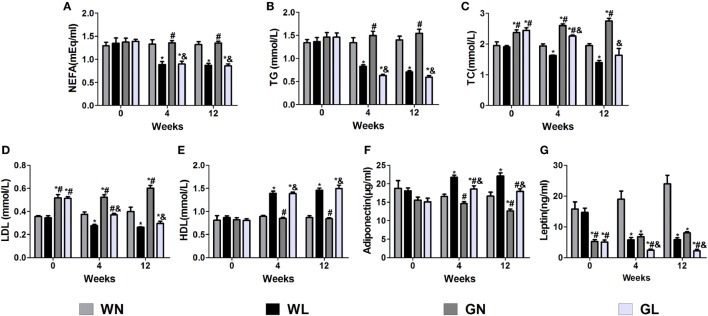
Liraglutide improved the lipid profiles and the leptin and adiponectin levels. **(A)** Non-esterified fatty acid (NEFA) levels. **(B)** Triglyceride (TG) levels.**(C)** Total cholesterol (TC) levels. **(D)** Low-density lipoprotein (LDL) cholesterol levels. **(E)** High-density lipoprotein (HDL) cholesterol levels. **(F)** Adiponectin levels. **(G)** Leptin levels. Data are presented as the mean ± SEM. *P < 0.05 vs WN group; ^#^*P* < 0.05 vs WL group; and ^&^*P* < 0.05 vs GN group.

Before intervention, there were no differences in the NEFA (Figure 3A), TG (Figure 3B), and HDL (Figure 3E) levels between Wistar and GK rats, while the TC (Figure 3C) and LDL (Figure 3D) levels of GK rats were drastically higher than those of Wistar rats. The adiponectin levels (Figure 3F) were also without difference between the two types of rats; however, the leptin levels (Figure 3G) were significantly higher in Wistar rats. After intervention for 4 weeks, the lipid profile levels and the adiponectin and leptin levels were significantly improved. Compared with the two control groups, liraglutide treatment for 12 weeks dramatically reduced the lipid profile levels, including NEFA, TG, TC, and LDL (all *P* < 0.05), and there were no differences between the WL and GL groups (Figures 3A–D). Contrarily, from the fourth week, liraglutide significantly elevated the HDL levels in both Wistar and GK rats (all *P* < 0.05). Interestingly, throughout the experiment, TG and NEFA levels of the WN group were always comparable to those of the GN group, while the TC and LDL levels of the GN group were gradually higher than those of the WN group. However, after liraglutide treatment, there was no difference in the lipid profile between the WL and GL groups. The dramatic increase in the adiponectin levels (Figure 3F) and the obvious reduction in leptin levels (Figure 3G) were also observed from the fourth week of intervention.

### Liraglutide Decreased Ra_glu_, GNG, the TG Content, and Synthetic Rates of TG in the Liver and Ra_gly_ but Increased Fatty Acid β-Oxidation Rates in the Skeletal Muscle

**Figure 4:**
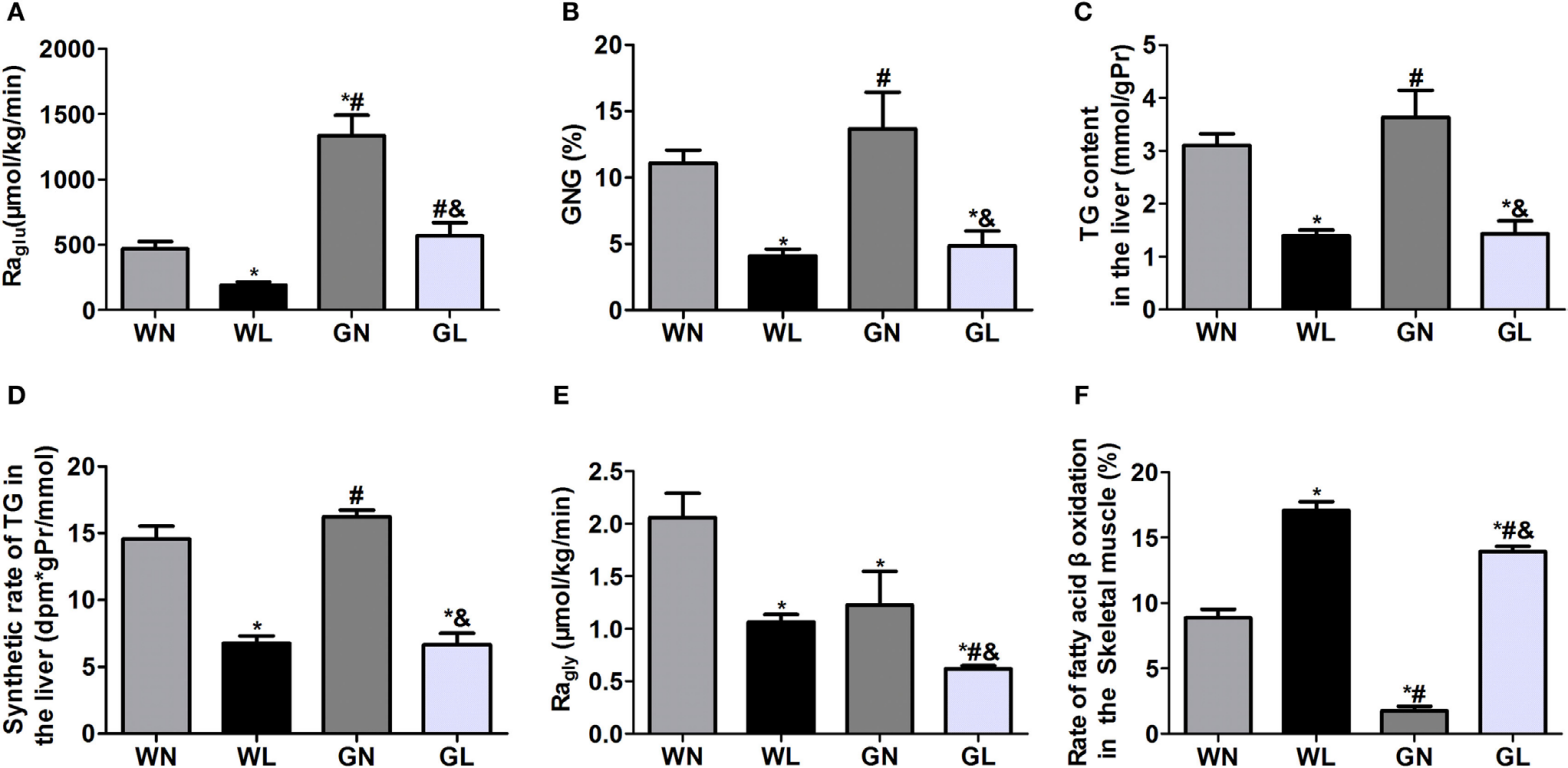
Liraglutide inhibited hepatic glucose production, decreased hepatic fat deposition and lipogenesis, and promoted fatty acid β oxidation. **(A)** Rate of glucose appearance (Ra_glu_). **(B)** Gluconeogenesis (GNG). **(C)** Triglyceride (TG) content in the liver. **(D)** TG synthetic rates in the liver. **(E)** Rate of glycerol appearance (Ra_gly_). **(F)** Fatty acid β-oxidation in the skeletal muscle. Data are presented as the mean ± SEM. *P < 0.05 vs WN group; ^#^*P* < 0.05 vs WL group; and ^&^*P* < 0.05 vs GN group.

The Ra_glu_ (Figure 4A) and GNG (Figure 4B) were considerably decreased in both the WL and GL groups after a 12-week liraglutide intervention. The TG content (Figure 4C) and synthetic rates of TG (Figure 4D) in the liver were also significantly reduced in the two intervention groups (all *P* < 0.05), and there were no significant differences between these two groups. The Ra_gly_ (Figure 4E), representing the extent of lipolysis, was also decreased in the two treatment groups (WL vs WN: 1.06 ± 0.20 vs 2.06 ± 0.61 μmol/kg/min; GK + LIRA vs GK + NS: 0.62 ± 0.07 vs 1.23 ± 0.78 μmol/kg/min, *P* < 0.05), whereas fatty acid β-oxidation (Figure 4F) was dramatically increased in both Wistar and GK rats (WL vs WN: 17.07 ± 1.48 vs 8.85 ± 1.63%; GL vs GN: 13.92 ± 0.94 vs 1.75 ± 0.78%, *P* < 0.05).

### Liraglutide Reduced Adipocyte Size in Both Visceral and Subcutaneous Fat Depots

**Figure 5:**
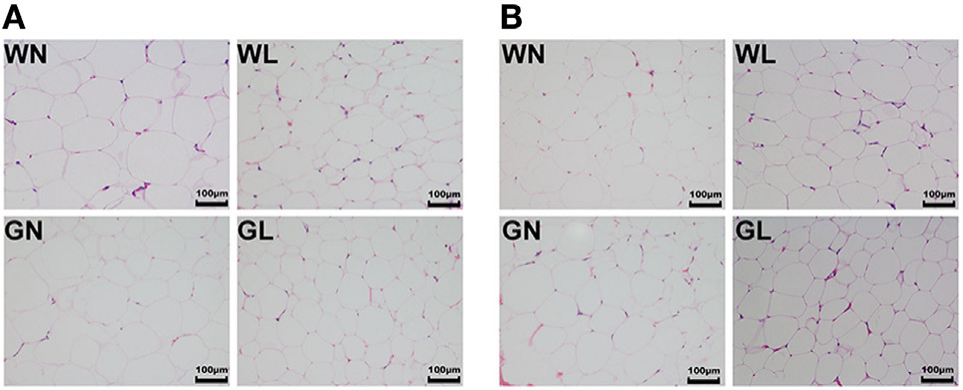
Liraglutide reduced adipocyte size in both visceral and subcutaneous fat depots. **(A)** Mesenteric white adipose tissues (WAT) hematoxylin and eosin (HE) staining (magnification 200×). **(B)** Inguinal WAT HE staining (magnification 200×).

Mesenteric WAT and inguinal WAT represented visceral and subcutaneous fat depots, respectively. After 12-week intervention, adipocyte sizes of mesenteric WAT were reduced by liraglutide in both Wistar and GK rats (Figure 5A). Similarly, compared with the corresponding control groups, liraglutide also decreased adipocyte sizes of inguinal WAT in both Wistar and GK rats (Figure 5B).

### Liraglutide Changed the Structure of Gut Microbiota in Both Simple Obese and Diabetic Obese Rats

**Figure 6:**
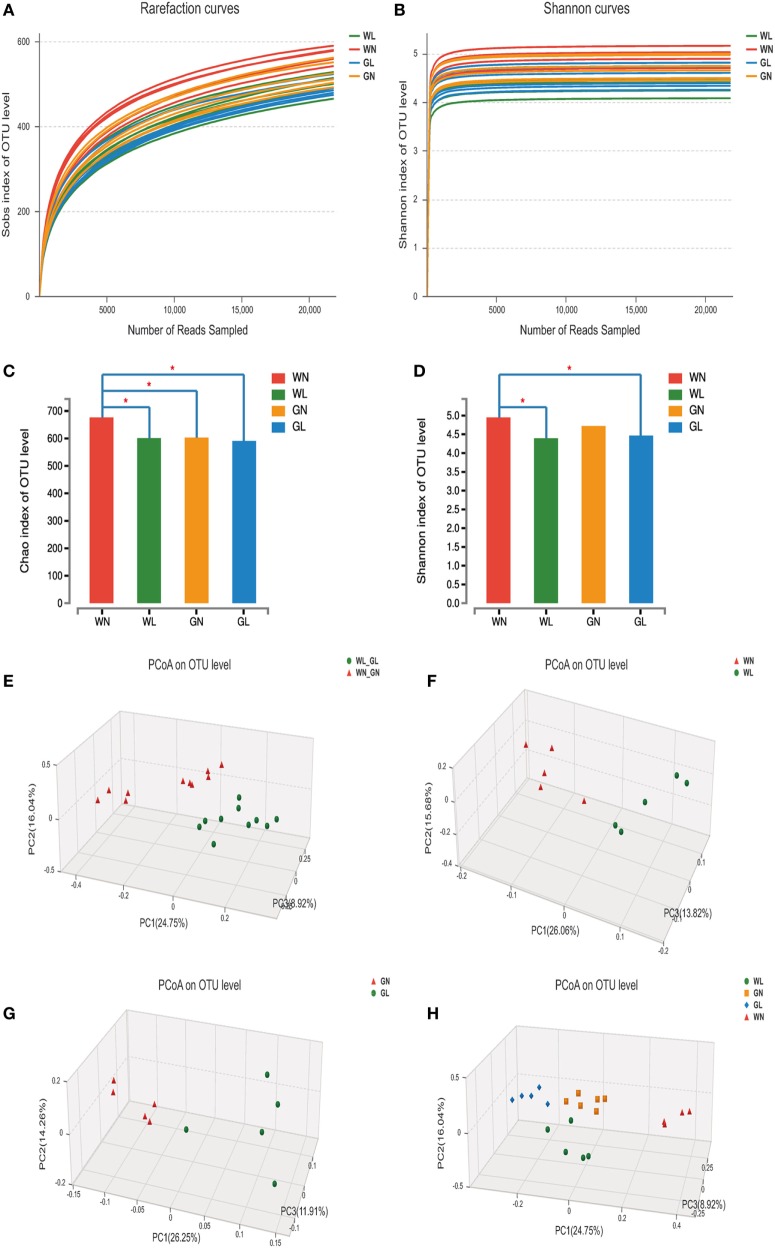
Liraglutide changed the overall structure of gut microbiota. **(A)** The rarefaction curves. **(B)** Shannon curves. **(C)** Chao index levels. **(D)** Shannon index levels. **(E)** Principal coordinate analysis (PCoA) generated using an unweighted UniFrac metric between before and after liraglutide intervention in all rats. **(F)** PCoA between WN and WL groups. **(G)** PCoA between GN and GL groups. **(H)** PCoA between the four groups.

A total of 1,975,595 valid reads were obtained from 20 samples and were delineated into 980 OTUs at similarity level cutoff of 97%. The rarefaction and estimator curves were shown in Figure 6. The rarefaction curves (Figure 6A) and Shannon curves (Figure 6B) tended to reach the saturation plateau, which indicated that the sequencing was deep enough to capture most of the OTUs within our samples. The Chao and Shannon indices of the WL group were significantly lower than those of the WN group. In GK rats, the Chao and Shannon indices also tended to be lower in the GL group than in the GN group, but there was no significant difference between these two groups (Figures 6C,D). This demonstrated that liraglutide treatment could result in a decrease in both the microflora community richness and diversity, seemingly independent of the glycemic status of the rats. In addition, the results showed that the WN group had a higher Chao index than did the GN group (*P* < 0.05), with a comparable Shannon index. However, after liraglutide treatment, there were no differences in the Chao and Shannon indices between the WL and GL groups. This means liraglutide reduced microflora community richness more obviously in simple obese rats (Wistar rats) than it did in diabetic rats (GK rats).

To explore whether the liraglutide-mediated reduction in body weight and glucose levels had relationship with the alteration in the gut microbiota, the overall microbial structure from all the rats was profiled. The PCoA score plots showed a substantial rearrangement of the microfloral structure in the liraglutide-treated rats compared with that of controls. In addition, the arrangement of the bacterial structure was different between Wistar and GK rats, indicating that the shift in gut microbiota was also related with the glucose levels (Figure 6E). The first three PCoA scores were 26.06, 15.68, and 13.82%, accounting for 55.56% of the variation between the WN and WL groups (Figure 6F). Similar results were found between the GN and GL groups: PC1, PC2, and PC3 were 26.25, 14.26, and 11.91%, respectively (Figure 6G). When the data from all samples were analyzed together, primary differences of microfloral structure could still be detected among the four groups (PC1/PC2/PC3: 24.75, 16.04, and 8.92%, respectively) (Figure 6H).

### Liraglutide Changed the Microbial Composition in Both Simple Obese and Diabetic Obese Rats

**Figure 7:**
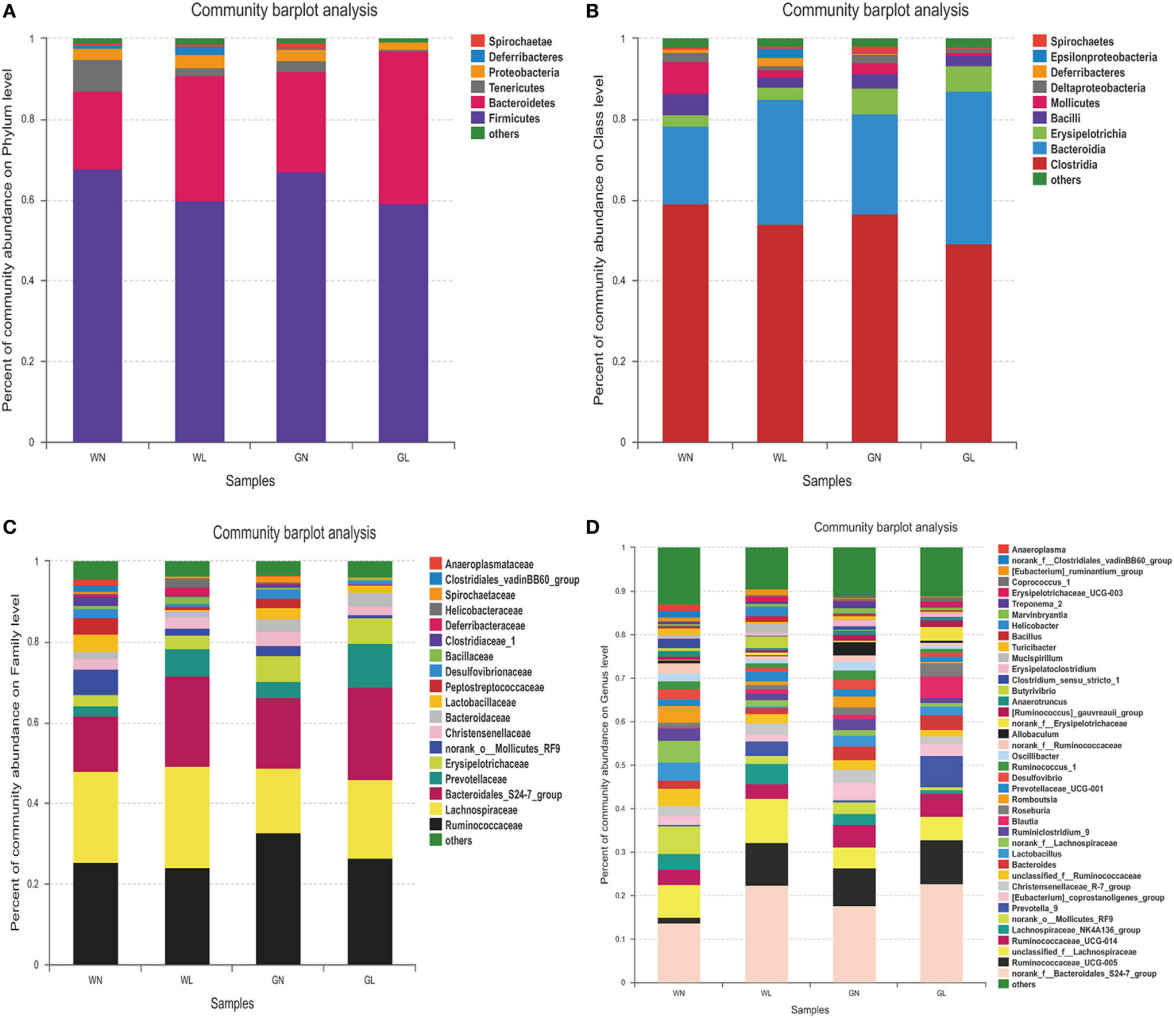

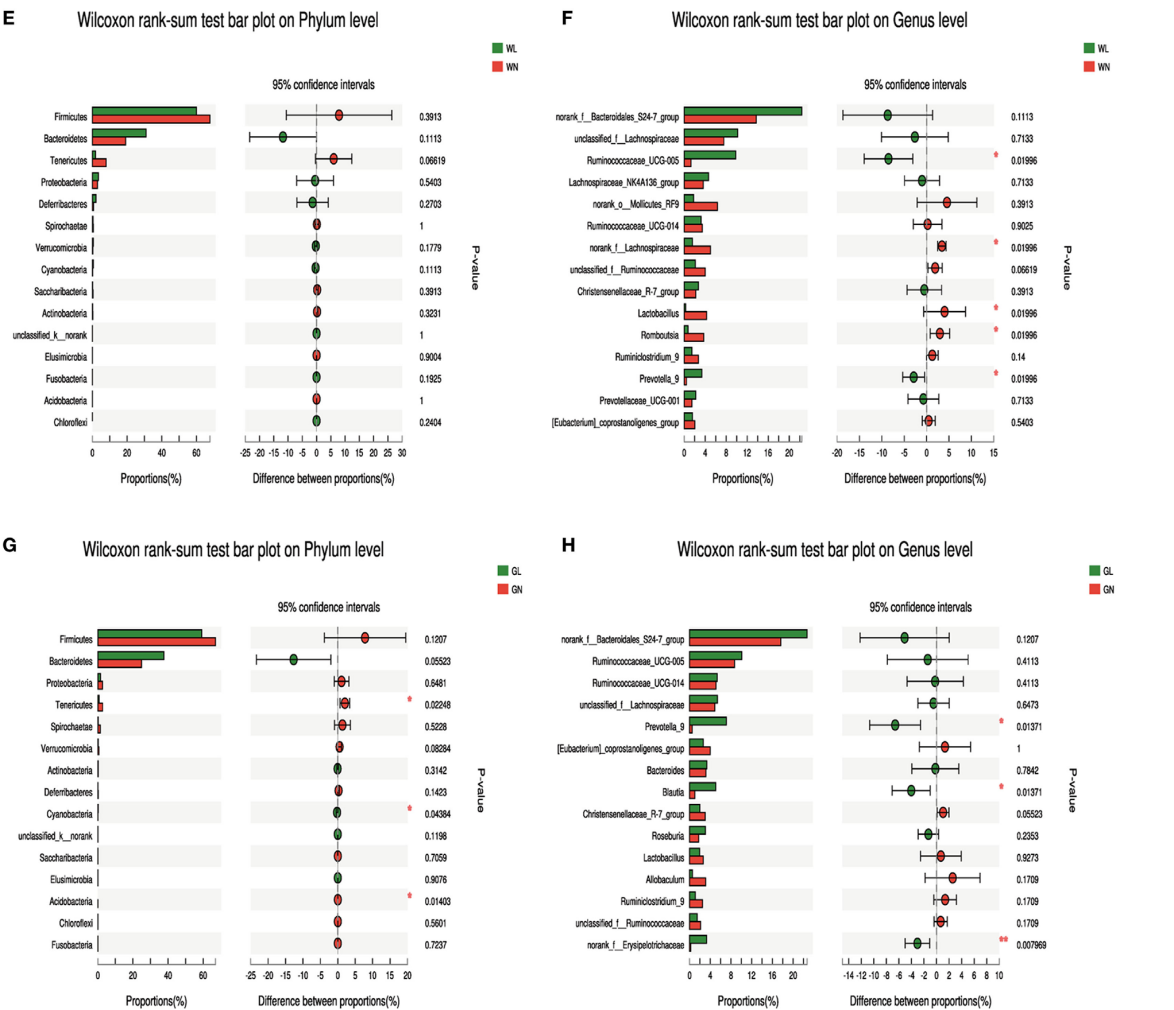
Liraglutide changed the composition of gut microbiota in both simple obese and type 2 diabetes mellitus obese rats. **(A)** Composition of gut microbiota at the phylum level. **(B)** Composition of gut microbiota at the class level. **(C)** Composition of gut microbiota at the family level. **(D)** Composition of gut microbiota at the genus level. **(E)** The differential species between the WN and WL groups at the phylum level. **(F)** The differential species between the WN and WL groups at the genus level. **(G)** The differential species between the GN and GL groups at the phylum level. **(H)** The differential species between the GN and GL groups in the genus level. *P < 0.05 and **P < 0.01.

The patterns seen in microbial composition were quite dissimilar between the liraglutide treatment groups and the corresponding control groups in both Wistar and GK rats. At the phylum level, the microflora of the four groups was dominated by species of the phyla *Firmicutes, Bacteroidetes, Tenericutes*, and *Proteobacteria*. Liraglutide obviously increased the *Bacteroidetes* in both Wistar and GK rats (WN vs WL: 0.19 ± 0.04 vs 0.31 ± 0.09; GN vs GL: 0.25 ± 0.06 vs 0.38 ± 0.09; *P* < 0.05) (Figure 7A). The abundance of *Firmicutes* also tended to be decreased in these two types of rats after liraglutide intervention, but there was no significant difference between them. This resulted in a higher *Bacteroidetes*-to-*Firmicutes* (B/F) ratio in the treatment groups than in the corresponding control groups (WN vs WL: 0.29 ± 0.07 vs 0.54 ± 0.22; GN vs GL: 0.38 ± 0.13 vs 0.67 ± 0.23; *P* < 0.05) (Figure 7A). At the class level, HFD resulted in a dramatically lower percentage of *Bacteroidia* (phylum *Bacteroidetes*) and a comparatively higher percentage of *Clostridia* (phylum *Firmicutes*) in both Wistar and GK rats, both of which were reversed by liraglutide treatment (WN vs WL: 0.19 ± 0.04 vs 0.31 ± 0.09, *P* < 0.05; 0.59 ± 0.07 vs 0.54 ± 0.18, *P* > 0.05; GN vs GL: 0.25 ± 0.06 vs 0.40 ± 0.05, *P* < 0.05; 0.57 ± 0.09 vs 0.49 ± 0.07, *P* > 0.05) (Figure 7B). At the family level, the total abundances of *Ruminococcaceae* and *Lachnospiraceae*, both belonging to the phylum *Firmicutes*, showed no difference among the four groups, but an obvious increase in *Bacteroidales* (phylum *Bacteroidetes*) was observed in the WL and GL groups compared with the corresponding controls (Figure 7C). As observed at the genus level, liraglutide led to marked changes of microbiota composition in the majority genus, with a significant enriching effects on *Bacteroidales* (phylum *Bacteroidetes*) (*P* < 0.05). The total levels of most genera from the phylum *Firmicutes* also showed no significant difference between the intervention and control groups (Figure 7D).

The differential species analysis (a taxonomy-based comparison) was also conducted between the liraglutide-treated group and controls in Wistar or GK rats. The results showed that the genera within the phylum *Bacteroidetes* were increased, while the genera within the phylum *Firmicutes* were decreased in Wistar rats (Figures 7E,F). Interestingly, at the phylum level, there were differences in phyla *Tenericutes, Cyanobacteria*, and *Acidobacteria* between the GN and GL groups (Figure 7G), but at the genus level, the genera mainly within the phyla *Bacteroidetes* and *Firmicutes* showed differences between these two groups (Figure 7H).

### Relationship Between Gut Microbiota Composition and Metabolic Parameters

**Figure 8:**
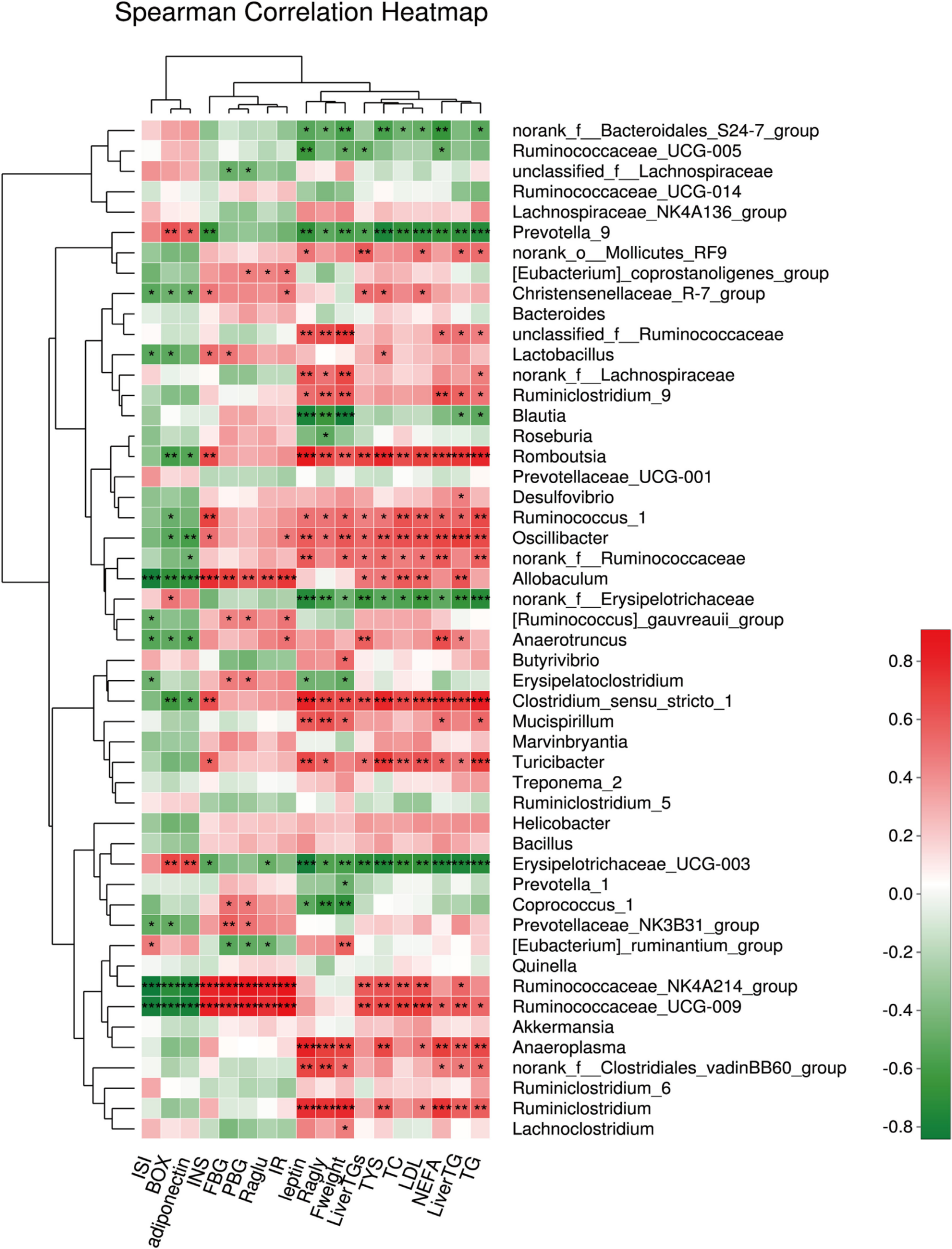
The relationship between microbiota composition and metabolic parameters. *P < 0.05, **P < 0.01, and ***P < 0.001.

We also assessed the relationship between the relative abundance of dominant bacterial genera and metabolic parameters to identify the genera that might contribute to the anti-hyperglycemia and anti-obesity effects. The results showed that the microbiota (such as *Romboutsia* and *Ruminiclostridium*), belonging to the phylum *Firmicutes*, were positively associated with the obesity-related parameters, including the lipid profile and lipogenesis in the liver, etc. In contrast, the microbiota (such as *Prevotella*.) belonging to the phylum *Bacteroidetes* correlated negatively with the obesity-related parameters (all *P* < 0.001). In addition, we found the microbiota *Ruminiclostridium* belonging to the phylum *Firmicutes* had a significantly positive relationship with hyperglycemia. In this study, genera having anti-hyperglycemia effects were not found (Figure 8).

## Discussion

In this study, we found that liraglutide improved both glucose and lipid metabolism *via* modulating the structure of gut microbiota. After intervention with liraglutide, the abundance and diversity of the gut microbiota were all decreased greatly. In Spearman’s correlation analysis, the correlation of the gut microbiota with metabolic parameters was also displayed.

Changes in gut microbiota composition and activity have been associated with different metabolic disorders, including obesity, diabetes, and cardiometabolic disorders (7, 20, 21). Previous studies displayed that the gut microbiota in obese subjects was characterized by higher populations of *Firmicutes* and lower populations of *Bacteroidetes*, as well as a reduction in microbial diversity (22). Several probiotics and their mixtures have been reported to improve metabolic syndrome (MS) (23, 24) by modulating the composition of the gut microbiota or its metabolites (23–25). For example, *Bifidobacterium animalis* ssp. *lactis* GCL2505 treatment significantly increased the abundance of phylum *Actinobacteria* but did not affect the *Bacteroidetes*/*Firmicutes* ratio (7). *B. lactis* GCL2505, a highly viable and proliferative probiotic, exerted anti-MS effects, such as improving glucose tolerance and suppressing visceral fat accumulation, *via* changing the overall structure of the gut microbiota (7). In addition, Li et al. (26) showed a simultaneous reduction of both *Firmicutes* and *Bacteroidetes* but a significant increase in *Proteobacteria* after the gastric bypass in a rat model. Nicola Basso et al. (17) found no significant changes in the *Firmicutes*/*Bacteroidetes* ratio after glandular gastrectomy but rather dramatic shifts in relative abundance within the *Firmicutes*, with increased relative abundance of *Lactobacillus*, and reduced abundance of *Ruminococcus*, characterizing improved metabolic health. All these suggested that modulating the structure of the gut microbiota was considered an emerging therapeutic strategy for improving metabolic disorders.

In this study, liraglutide increased the *Bacteroidetes*-to-*Firmicutes* ratio to lower weight significantly regardless of the glycemic status, consistent with the results of previous studies (14, 15, 27). GLP-1 receptor agonists, mimicking gut-derived molecules, have been used for diabetes and obesity treatment (1). Previous reports indicated that the proportions of the phylum *Firmicutes* and the class *Clostridia* were significantly reduced in the guts of diabetic patients (28). The exact correlation between the *Firmicutes*/*Bacteroidetes* ratio and obesity remain under discussion. Some studies indicated an increased ratio (22, 29), while others indicated the inverse (6), and recent studies have revealed no correlation at all (30, 31). Lin Wang et al. observed no substantial changes in the abundance of the phyla *Firmicutes* and *Bacteroides* but found a higher *Firmicutes*-to-*Bacteroidetes* ratio under liraglutide administration (4). Our study found the abundances of *Bacteroides* were significantly increased and the abundances of *Firmicutes* tended to be decreased after liraglutide treatment. This discrepancy may be attributed to the different model systems used (mice vs rats), different lifestyles (such as under field conditions without restriction, intensity and regularity of exercise as well as total daily energy intake) and methodological differences in DNA extraction protocols as well as primer design (6). When these factors were excluded in well-defined experiments in both animals and human studies (5, 22, 29), the results showed the positive relationship between the *Firmicutes*-to-*Bacteroidetes* ratio and obesity. However, when the study was conducted in human volunteers under field conditions without restriction, the finding was contrary (6). The exact reasons still need further investigation.

Liraglutide mainly changed the structure of gut microbiota at the family- and genus-levels, which might be more relevant to body weight. The genera *Candidatus, Arthromitus* (16), *Roseburia* (32), and *Marvinbryantia* (33) may contribute to weigh gain, while the genera *Lactobacillus* (34) and *Coprococcus* (35) are known to be associated with weight loss. Previous studies demonstrated that liraglutide decreased the genera *Roseburia, Marvinbryantia, Erysipelotrichaceae Incertae Sedis*, and *Parabacteroides* (obesity-related phylotypes) while enriching genera *Blautia* and *Coprococcus* (lean-related phylotypes) (4). This study showed that liraglutide also decreased the obesity-related phylotypes (such as *Romboutsia, Ruminiclostridium*, and *Erysipelotrichaceae*) and increased the lean-related phylotypes (such as *Prevotella*, etc.) in both simple obese and T2DM obese rats. This further confirmed that the weight-controlling effect of liraglutide was independent the glycemic status.

How did liraglutide modulate the structure of gut microbiota to prevent the weight gain? One possible mechanism is its effects on incretin augmentation. As a GLP-1RA, liraglutide increased the level of active GLP-1 to 60–90 pmol/L *via* subcutaneous administration (6 µg/kg, once daily) in humans (36), which then greatly delayed the gastric emptying rate and gut transit time and potentially affected the gut lumen internal environment (e.g., the local pH value and nutrient composition). All these factors are known to affect the composition of the microbiota. In addition, liraglutide can cross the blood–brain barrier and had been found to bind to neurons within the arcuate nucleus and other sites within the hypothalamus (37). GLP-1 receptors are located on neurons innervating the portal vein (38), on β cells of the pancreas (39), in the central nervous system (40), and on vagal afferents that innervate the gut in proximity to L cells (41). Once released, GLP-1 can act locally on afferent neurons innervating the gastrointestinal tract which signal to the caudal brainstem or enteric neurons, and/or they can enter the circulation to act centrally, or on peripheral targets, to regulate metabolic disorders (42). These indicated a possible gut–brain axis for mediating weight-loss and glucose-controlling effects of liraglutide (1). However, the exact mechanisms still require more investigations.

Our study has some novel insights. First, we explored the relationship between the metabolic effects of liraglutide and the structural changes of gut microbiota in both simple obese and T2DM obese rats. Second, except for exploring the conventional correlation of metabolic parameters with microbiota, we also detected the association of HGP (Ra_glu_ and GNG) and lipid metabolism indices (fat deposition, lipogenesis, lipolysis, and fatty acid oxidation) with microbiota. However, there are also some limitations. First, the doses of liraglutide were not gradient, so we could not observe whether changes of gut microbiota were dependent of the dosage of liraglutide. Second, our rats were not fed in germ-free environment. Finally, fecal samples in our study were not representative of the entire intestine. Since altered small and large intestinal microbial composition might have different influence on host metabolism, it was necessary to obtain fecal samples in different intestinal segments. Thus, further investigations are needed.

## Conclusion

Our study provided evidence that liraglutide could prevent weight gain *via* modulating the gut microbiota composition, including inhibition of microbiota abundance and diversity, elevation of the B/F ratio, and an argument for the lean-related microbial phenotypes in both simple obese and T2DM obese subjects. However, much more in-depth analysis is necessary to elucidate how and to what extent the weight-controlling effects of liraglutide are dependent on the gut microbiota modulation.

## Ethics Statement

This study was carried out in accordance with the recommendations of the ethical principles in animal research adopted by the Department of Laboratory Animal Science, Shanghai JiaoTong University School of Medicine, Shanghai, China and The protocol was approved by the Animal Experimental Ethical Committee of Shanghai JiaoTong University School of Medicine, Shanghai, China.

## Author Contributions

LZ and YC performed the experiment, collected the data, and drafted the manuscript. FX, BA, and WZ performed the experiment and performed the statistical analysis. YL, YG, and NW conceived the study and participated in its design and coordination and helped to draft the manuscript. All the authors read and approved the final manuscript.

## Conflict of Interest Statement

The authors declare that the research was conducted in the absence of any commercial or financial relationships that could be construed as a potential conflict of interest.

## Footnotes

^1^http://drive5.com/uparse/.

^2^http://rdp.cme.msu.edu/.

## Abbreviations

GLP-1RA, glucagon-like peptide-1 receptor agonists; FBG, fasting blood glucose; PBG, postprandial blood glucose; FINS, fasting insulin; HOMA-IR, homeostatic model assessment-insulin resistance; ISI, insulin sensitivity index; TG, triglyceride; TC, total cholesterol; LDL, low-density lipoprotein; NEFA, non-esterified fatty acid; Ra_glu_, rate of glucose appearance; GNG, gluconeogenesis; Ra_gly_, rate of glycerol appearance; T2DM, type 2 diabetes mellitus; AUC, area under the curve; IPGTT, intraperitoneal glucose tolerance test; IPITT, intraperitoneal insulin tolerance test.
